# Eclipse-Related Emergency Ophthalmology Presentations Following the August 2026 Solar Eclipse in the United Kingdom

**DOI:** 10.3390/vision10040071

**Published:** 2026-09-24

**Authors:** Khawaja Muhammad Ammar Ali Javed, Emer Chang, Hanif Suleman, Xiaoli Chen, Naduviledeth Ajith Kumar, Fizza Mushtaq, Saaeha Rauz, Yu Jeat Chong

**Affiliations:** 1 Academic Unit of Ophthalmology, Department of Inflammation and Ageing, School of Infection, Inflammation, and Immunology, College of Medicine and Health, University of Birmingham, Birmingham B18 7QH, UK; 2 Birmingham and Midland Eye Centre, City Health Campus, Sandwell and West Birmingham NHS Trust, Birmingham B18 7QH, UK

**Keywords:** solar retinopathy, eclipse retinopathy, solar eclipse, emergency ophthalmology, public health, health-seeking behaviour, optical coherence tomography

## Abstract

**Introduction:** Solar eclipses can cause solar retinopathy, but eclipse-related presentations may reflect public concern as well as true retinal injury. We evaluated the immediate ophthalmic service impact of the 12 August 2026 solar eclipse in the United Kingdom and characterised associated clinical, imaging, and online search findings. **Methods:** An observational case series was conducted at Birmingham and Midland Eye Centre, a tertiary eye casualty unit serving Birmingham and the wider West Midlands, England. Patients attending on 13 August 2026 with new visual symptoms or concern about ocular injury after viewing the Sun or eclipse were identified. Clinical characteristics, viewing behaviour, presenting and pinhole visual acuity, and multimodal retinal imaging findings were recorded, including optical coherence tomography, colour fundus photography, and fundus autofluorescence. Google Trends was used to assess contemporaneous UK online search interest in eclipse- and eye-related terms. **Results:** Of 128 eye casualty attendances on 13 August 2026, 10 (7.8%) were eclipse-related. Median age was 37.5 years, 7/10 (70%) patients were female, and 9/10 (90%) reported bilateral symptoms. Seven patients (70%) intentionally viewed the Sun or eclipse, and none reported confirmed use of certified eclipse glasses. Reduced or blurred vision was reported by 5/10 (50%) and photophobia by 4/10 (40%). Visual acuity was 6/9 or better in all 20 eyes and 6/6 or better in 17/20 (85%). Pinhole visual acuity was unchanged from presenting visual acuity in all 20 eyes. No clinically apparent macular abnormality or characteristic acute solar-retinopathy phenotype was identified. Google Trends showed a sharp, temporally concentrated increase in eclipse-related ocular searches around the eclipse, with several post-exposure queries showing marked increases or being classified as Breakout. **Conclusions:** The eclipse produced an immediate measurable burden on emergency ophthalmology services, with approximately 1 in 13 attendances on the following day being eclipse-related despite no definite acute solar retinopathy being identified. The accompanying increase in online search activity suggests substantial public concern with little demonstrable acute retinal pathology. As surveillance was restricted to the first full day after the eclipse, later presentations and longer-term symptom evolution cannot be excluded.

## 1. Introduction

In Benjamin Jowett’s translation of Plato’s *Phaedo*, Socrates warns of the danger of looking directly at the sun: “Now there is a danger in the contemplation of the nature of things, as there is a danger in looking at the sun during an eclipse, unless the precaution is taken of looking only at the image reflected in the water, or in a glass [1].”

Solar eclipses are rare, predictable events that generate considerable public attention. Direct solar viewing can cause solar retinopathy, a photochemical macular injury concentrated on the foveal photoreceptors and retinal pigment epithelium (RPE) [2]. Patients can report symptoms of central blur, scotomata, metamorphopsia, and photophobia. Solar retinopathy (SR) is not confined to eclipses and has also been described following deliberate or prolonged sungazing, including religious practices and other forms of unprotected direct solar observation [3].

On 12 August 2026, a deep partial solar eclipse was visible across the United Kingdom (UK) and Ireland, with approximately 90–96% of the solar disc obscured, depending on location. Maximum eclipse in Birmingham, England occurred at approximately 19:11 British Summer Time (BST). This represented the greatest solar obscuration visible from the UK and Ireland since 1999, and the next total solar eclipse visible from the UK is not expected until 2090 [4]. Viewing conditions were also relevant to the potential for direct solar exposure. Contemporaneous forecasts indicated generally favourable conditions across much of central and southern England. Although some cloud was expected across the West Midlands, conditions were anticipated to permit at least intermittent viewing of the eclipse [5].

Substantial safety messaging preceded the event. The UK Health Security Agency (UKHSA) advised that ordinary sunglasses were inadequate and recommended certified solar viewers or indirect viewing methods [6]. Two days before the event, the Royal College of Ophthalmologists (RCOphth) similarly warned that even brief unprotected exposure could cause lasting central visual damage and advised prompt eye-care assessment if a post-exposure change in vision occurred [7].

Certified eclipse viewers conforming to ISO 12312-2 are specifically designed for direct solar observation by attenuating ultraviolet, visible, and infrared solar radiation to safe viewing levels; ordinary sunglasses, regardless of darkness, do not provide equivalent protection [8]. The College of Optometrists similarly advised that ordinary sunglasses were inadequate for direct solar viewing and recommended specially designed solar-filter glasses bearing appropriate UKCA or CE markings. For individuals unable to obtain eclipse glasses, indirect viewing using a pinhole projector was recommended as a safe alternative. Local availability or distribution of certified eclipse viewers through pharmacies or eye-care practices was not assessed in this study [9].

The ophthalmic consequences of eclipse viewing have been documented repeatedly, and presentations may substantially outnumber confirmed injuries. In this context, eclipse retinopathy refers to solar retinopathy that occurs after directly viewing the sun during a solar eclipse. Following the 1999 UK total eclipse, Michaelides et al. undertook national active case ascertainment and identified 70 cases of eclipse-related visual loss: 84% had an abnormal macular appearance at presentation, but no continued visual loss or symptoms were reported at 6 months [10]. In Leicester, UK, Wong et al. prospectively recorded 45 eclipse-related eye casualty attendances, of whom 20 were symptomatic and five had visible retinal changes; four remained symptomatic at 7 months [11].

These early studies largely relied on fundus examination and colour fundus photography (CFP) to characterise the damage caused by eclipse retinopathy. Within the first 7 days, a yellow cystic macular lesion and/or altered foveal reflex was seen on CFP [10,11]. At 1 month follow up, this lesion may clear completely or remain as a white area of RPE hypopigmentation [10].

One of the most remarkable reports of solar retinopathy is that of Hope-Ross et al. (1993), who studied the retina of a 65-year-old man whose eye was already scheduled for enucleation because of choroidal melanoma [12]. Before surgery, the patient deliberately stared at the sun and developed typical solar retinopathy, allowing histological examination just six days later. Ultrastructural analysis showed marked photoreceptor and retinal pigment epithelial damage, while the relative resistance of foveal cones was proposed to explain the generally favourable visual prognosis.

More recent eclipse retinopathy reports include multimodal imaging with optical coherence tomography (OCT) and fundus autofluorescence (FAF). OCT findings have demonstrated that photochemical injury in eclipse retinopathy appears to primarily affect the outer retina, with the following biomarker seen within the first few weeks: curvilinear column of hyperreflective Henle fibres [13,14,15]; loss/hyporeflective ellipsoid zone (EZ) [13,16,17]; loss/hyporeflective interdigitation zone (IZ) [13,14]; intraretinal cyst [18,19]. In some cases, all layers of foveola were affected [16]. Although visual acuity (VA) often improves, persistent scotomata or structural abnormalities have been described from months to years after exposure [20].

On FAF, hyperautofluorescence of the foveola has been described in the early phase of eclipse retinopathy, suggestive of increased macular inflammation and foveal metabolism as a possible self-repair response [19].

A separate consideration is that the healthcare footprint of an eclipse may reflect heightened symptom vigilance as much as phototoxic injury, and that this concern may be measurable outside clinical settings. Waisberg et al. described a marked Google search spike for “my eyes hurt” during the April 2024 North American eclipse [21], whereas a large US emergency-department analysis found no statistically significant post-eclipse rise in coded ocular injury presentations [22].

We therefore undertook a case series of eclipse-related presentations to a tertiary eye casualty department immediately after the 12 August 2026 eclipse. As the last comparable UK event occurred in 1999, before OCT and FAF entered routine clinical use, the 2026 eclipse provided an opportunity to characterise a UK eclipse-exposed cohort using contemporary multimodal imaging. Our primary aim was to quantify the immediate service burden. Secondary aims were to characterise viewing behaviour, symptoms, and multimodal imaging findings; determine whether definite acute solar retinopathy was evident on day 1; and examine online search activity around the time of the eclipse as a contextual measure of public concern.

## 2. Materials and Methods

This observational case series included consecutive patients presenting to Birmingham and Midland Eye Centre (BMEC) eye casualty on 13 August 2026 with new ocular or visual symptoms, or concern about ocular injury, after viewing the Sun or eclipse. BMEC is a supra-regional tertiary ophthalmic centre serving Birmingham and the wider West Midlands in England and receiving tertiary referrals from elsewhere in the Midlands; consequently, its eye casualty service does not have a single geographically bounded catchment population. The project had been prospectively registered locally as a clinical effectiveness audit at Sandwell and West Birmingham NHS Trust (Reference 3532), used routinely collected clinical data and did not alter patient care. Formal research ethics approval was not required under the institutional audit determination.

The day after the eclipse, eye casualty clinicians were asked to identify patients attending on 13 August 2026 with new visual symptoms, or concern about eye injury, after viewing the Sun or the eclipse. The principal service-impact analysis focused on 13 August 2026, the first full day after the eclipse.

The total number of patients attending BMEC eye casualty on 13 August 2026 was obtained from routine service data. Eclipse-related presentations were expressed as a proportion of all attendances. This measure describes the service footprint of eclipse-related concern and should not be interpreted as population incidence.

### 2.1. Data Collection

A standardised dataset captured age, sex, laterality, viewing intention, approximate exposure duration, use of protective eye equipment, symptom onset, presenting visual acuity (VA), pinhole VA, and retinal imaging findings. Exposure duration was recorded as <5 s, 5–30 s, 31–60 s, or >1 min. Pinhole VA was assessed in all patients. This study was designed to characterise acute presentations on the first full day following the eclipse and did not systematically capture subsequent symptom duration or longitudinal symptom evolution.

### 2.2. Imaging Modalities

Macular OCT scans were acquired using the SPECTRALIS OCT system (Heidelberg Engineering GmbH, Heidelberg, Germany) with the fast 25 B-scan protocol. Ultra-widefield CFP (pseudocolour) and FAF were performed using non-contact 200-degree retinal imaging (optomap; Optos plc, Dunfermline, UK). Definite day-1 solar retinopathy required a compatible exposure history together with a characteristic acute foveal phototoxic pattern on multimodal imaging. All available day-1 images were independently reviewed by three consultant ophthalmologists, who agreed on the presence or absence of acute solar retinopathy in each case.

CFPs were analysed for the presence of yellow, reddish-brown, or grey foveal/parafoveal lesions and an altered foveal reflex. FAF scans were analysed for the presence of foveolar hyperautofluorescence. Macular OCT scans were analysed for the following changes: Henle fibre layer hyperreflectivity; loss, hyporeflectivity, or disruption of the EZ and IZ; RPE disruption; intraretinal cysts; and foveolar changes. Where previous OCT, CFP, or FAF imaging was available, comparisons were made to assess for any interval change.

### 2.3. Online Search Interest (Google Trends)

Online public interest was explored using Google Trends (Google LLC, Mountain View, CA, USA; web-based platform, accessed 15 August 2026). On 15 August 2026, United Kingdom Web Search activity was examined over 12–13 August 2026 using six lay and eclipse-specific search terms entered simultaneously: “eyes hurt”, “eye damage”, “looked at sun”, “eclipse eyes”, “eclipse eye damage”, and “eyes hurt eclipse”. Interest over time was recorded on Google Trends’ normalised 0–100 scale, on which 100 represents peak relative popularity within the selected comparison. Top and rising related queries were additionally reviewed for “eyes hurt”. Google Trends reports relative search interest rather than absolute search counts; these data were therefore used only as contextual evidence of temporal information-seeking and public concern, not as a surrogate for ocular injury or clinical incidence [23]. A geographically restricted Birmingham analysis was explored but could not be performed because city-level geographic restriction was not available for the selected Google Trends analysis.

### 2.4. Statistical Analysis

Given the small event-specific cohort, analyses were descriptive. Continuous variables are reported as median and interquartile range (IQR), and categorical variables as number and percentage. VA is recorded in the Snellen format. All analyses were conducted using R version 4.5.1 (R Foundation for Statistical Computing, Vienna, Austria) [24].

## 3. Results

On 13 August 2026, 128 patients attended BMEC eye casualty. Across the four preceding Thursdays, median eye casualty attendance was 133 patients (IQR 128.8–135.3; range 119–139), compared with 128 attendances on 13 August 2026. Thus, overall attendance on the first full day after the eclipse was within the recent usual range. Ten presentations were eclipse-related, accounting for 7.8% of all attendances, or approximately 1 in 13 patients seen that day (Figure 1). All 10 presented between 09:58 and 18:03 on the first full day after the eclipse.

The median age was 37.5 years (IQR 29.8–42.5; range 21–60), and 7/10 (70%) were female. Nine of 10 (90%) described bilateral symptoms and 7/10 (70%) reported intentionally looking at the Sun or eclipse.

Six of 10 (60%) estimated direct viewing for <5 s, 2/10 (20%) for 5–30 s, 1/10 (10%) for 31–60 s and 1/10 (10%) for >1 min. Eight of 10 (80%) described a single sustained gaze and 2/10 (20%) repeated glances. None reported confirmed use of certified eclipse glasses. One patient attempted to protect the eyes by wearing three pairs of ordinary sunglasses simultaneously; another used a homemade cardboard-framed viewing device of uncertain certification.

Reduced or blurred vision was the most common symptom, reported by 5/10 patients (50%), followed by photophobia in 4/10 (40%); dyschromatopsia and floaters were each reported by 3/10 (30%). Other symptoms included dark spots or scotomata, metamorphopsia and ocular discomfort. Symptom onset was immediate in 9/10 (90%) and within 6–24 h in 1/10 (10%). VA was preserved: all 20 eyes were 6/9 or better and 17/20 (85%) were 6/6 or better. Pinhole VA was unchanged from presenting VA in all 20 eyes. Dilated examination showed no clinically apparent macular abnormality in any eye; one patient had vitreous floaters.

Macular OCT was performed in all 10 patients, while ultra-widefield CFP and FAF were each obtained in 9/10. No patient demonstrated a characteristic acute solar-retinopathy phenotype.

The above findings are summarised in Table 1.

Google Trends demonstrated a sharp, temporally concentrated surge in eclipse-related ocular searching on 12 August 2026 (Figure 2). Among the six predefined search terms, ‘eclipse eyes’ reached the maximum normalised relative-interest value of 100, with concurrent increases across the other eclipse- and eye-related terms. Eclipse-specific phrases predominated among the top queries associated with ‘eyes hurt’ (Table 2): ‘eyes hurt eclipse’, ‘eclipse eyes hurt’, and ‘eclipse hurt eyes’ had relative search-interest values of 100, 98, and 96, respectively. ‘Eyes hurt after eclipse’ increased by 650% and ‘my eyes hurt eclipse’ by 100%. Multiple explicitly post-exposure searches, including ‘eyes hurt after solar eclipse’, ‘eyes hurt after looking at eclipse’, and ‘eyes hurt after looking at sun’, were classified as Breakout queries.

## 4. Discussion

We identified that 10 of 128 (7.8%) eye casualty attendances on 13 August 2026 were related to the solar eclipse on 12 August 2026. Notably, total eye casualty attendance on 13 August was within the range observed over the four preceding Thursdays. The eclipse therefore appears to have altered the composition of emergency ophthalmic workload rather than increasing overall attendance volume, with approximately 1 in 13 presentations that day being eclipse-related. The key finding was that none of these patients demonstrated definite acute solar retinopathy. Eclipse-related concern therefore produced an immediate and considerable emergency ophthalmology footprint despite no definite acute SR.

The 1999 UK studies documented genuine retinal morbidity but were conducted before spectral-domain OCT and FAF were routinely available [10,11]. In the present cohort, OCT was performed in every patient and CFP/FAF in almost all, allowing a more sensitive structural assessment for subtle outer-retinal injury. Despite this, no characteristic acute solar retinopathy phenotype was identified. Similarly, the 2017 Utah experience showed that only a minority of patients presenting with visual concerns after the eclipse had findings consistent with solar retinopathy [17]. Together, these observations reinforce that the burden of presentation should not be assumed to reflect the burden of retinal injury. The presence of subjective symptoms despite preserved VA and absence of a characteristic structural phenotype is not necessarily contradictory. Transient photoreceptor adaptation or subtle photochemical dysfunction may produce visual disturbance without abnormalities detectable on routine structural imaging, while some reported complaints, particularly floaters and ocular discomfort, are nonspecific and may arise independently of macular phototoxicity. Formal microperimetry, contrast-sensitivity testing, and detailed colour-vision testing were not systematically performed, and subtle functional disturbance therefore cannot be excluded. Heightened symptom vigilance following a highly publicised exposure may also have contributed to presentation. The subsequent duration and evolution of symptoms were outside the predefined scope of this acute case series.

Characteristic acute SR typically comprises a small yellow or yellow-white foveal/parafoveal lesion or altered foveal reflex, while OCT may demonstrate acute outer-retinal or Henle-layer hyperreflectivity followed by disruption of the ellipsoid and interdigitation zones, sometimes with RPE disturbance or focal cystic/outer-retinal defects [3,14,19]. None of these characteristic patterns was identified in our cohort.

The contemporaneous Google Trends surge provides complementary evidence of heightened public concern around the eclipse. Eclipse-specific phrases dominated the top related queries, while multiple searches explicitly describing ocular symptoms following eclipse or solar viewing showed marked increases or were classified as Breakout. This supports a substantial population-level information-seeking response around the event. However, Google Trends remains an ecological signal and cannot establish that individual searchers had ocular symptoms, viewed the Sun, or subsequently sought medical care; values are normalised rather than absolute search volumes [23].

The exposure histories in our patient cohort suggested a related public-health issue: awareness of the need for protection did not always translate into use of an appropriate solar filter. One patient wore three pairs of ordinary sunglasses simultaneously, while another used a homemade cardboard-framed viewer whose certification could not be confirmed. This occurred despite explicit UKHSA and RCOphth guidance [6,7]. The extent of local access to certified eclipse viewers was not measured. Contemporary guidance explicitly anticipated that some members of the public might not have obtained eclipse glasses and recommended indirect viewing methods such as pinhole projection in that circumstance. Consequently, absence of certified protection in this cohort cannot distinguish limited access from lack of awareness or incorrect implementation of otherwise clear guidance. Given the small number of cases, no conclusions can be drawn from these behaviours regarding the effectiveness of existing guidance. Rather, these individual examples illustrate that awareness of recommended precautions does not necessarily ensure their correct implementation.

Strengths include capture immediately after a predictable exposure event at a tertiary eye casualty department, inclusion of all eclipse-related presentations rather than only patients with confirmed retinal injury, and systematic contemporary multimodal retinal imaging. This allowed assessment of service impact that would be missed by a confirmed-disease-only case series. Contemporaneous Google Trends data provided complementary contextual information regarding public information-seeking.

This study has several limitations. It was a single-centre case series of patients attending a supra-regional tertiary eye casualty service and therefore reflects healthcare utilisation rather than population incidence. Its findings may not be directly transferable to settings with different demographic or socioeconomic characteristics, levels of public-health awareness, access to certified solar-viewing equipment, or patterns of healthcare access. Patients may also have attended community optometrists, other ophthalmic units, general emergency departments, or primary care, while others may not have sought medical attention. Surveillance was restricted to the first full day after the eclipse; solar-retinopathy presentations may occur over the subsequent several days, particularly where individuals initially wait for spontaneous symptom resolution, and our study may therefore underestimate later attendances or retinal injury. This study did not systematically capture subsequent symptom duration or evolution. Exposure duration and protective behaviour were self-reported and subject to recall bias. Direct solar illuminance or spectral irradiance was not measured at the time and location of individual exposures. The eclipse occurred during the evening, and retinal exposure would have been influenced by solar elevation, atmospheric path length, and cloud cover; reported viewing duration alone therefore cannot quantify retinal radiant exposure. Finally, Google Trends data were analysed at UK level and cannot be directly matched geographically to the local BMEC cohort. A Birmingham-specific analysis could not be performed because city-level geographic restriction was not available for the selected analysis, and inferred online location may additionally be affected by IP-based geolocation and VPN use.

## 5. Conclusions

The 12 August 2026 solar eclipse produced an immediate and measurable impact on emergency ophthalmology services at BMEC, with approximately 1 in 13 eye casualty attendances on the following day being eclipse-related. VA was preserved and no definite acute solar retinopathy was identified. Alongside the contemporaneous surge in eclipse- and eye-related online searches, these findings suggest substantial public concern with little demonstrable acute retinal pathology. As surveillance was restricted to the first full day following the eclipse, later presentations cannot be excluded, and the longer-term evolution of symptoms was outside the scope of this study.

## Figures and Tables

**Figure 1:**
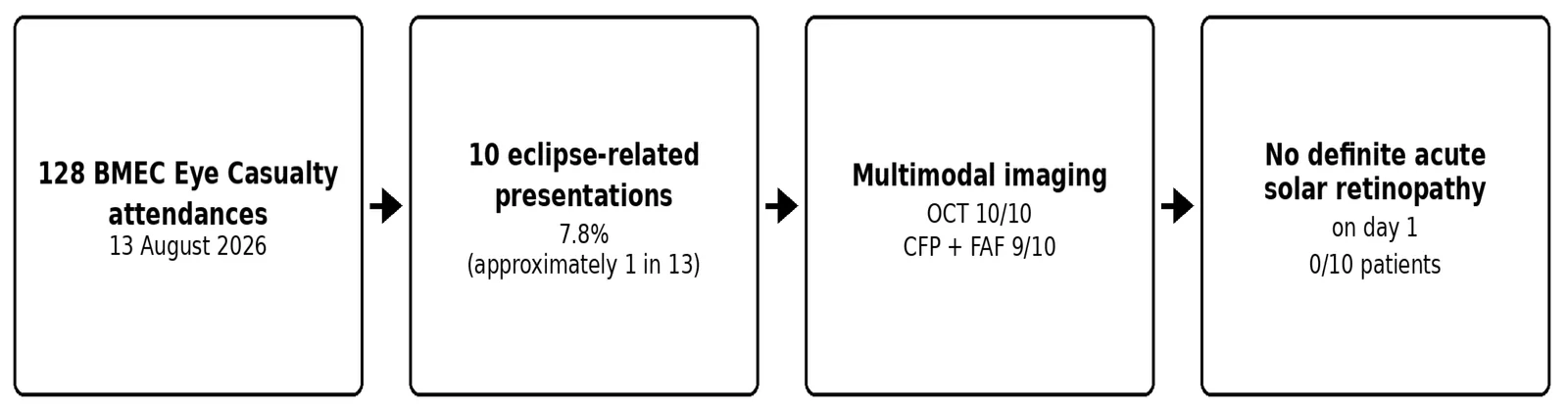
**Immediate service footprint of eclipse-related presentations on 13 August 2026.** CFP, colour fundus photography; FAF, fundus autofluorescence; OCT, optical coherence tomography.

**Figure 2:**
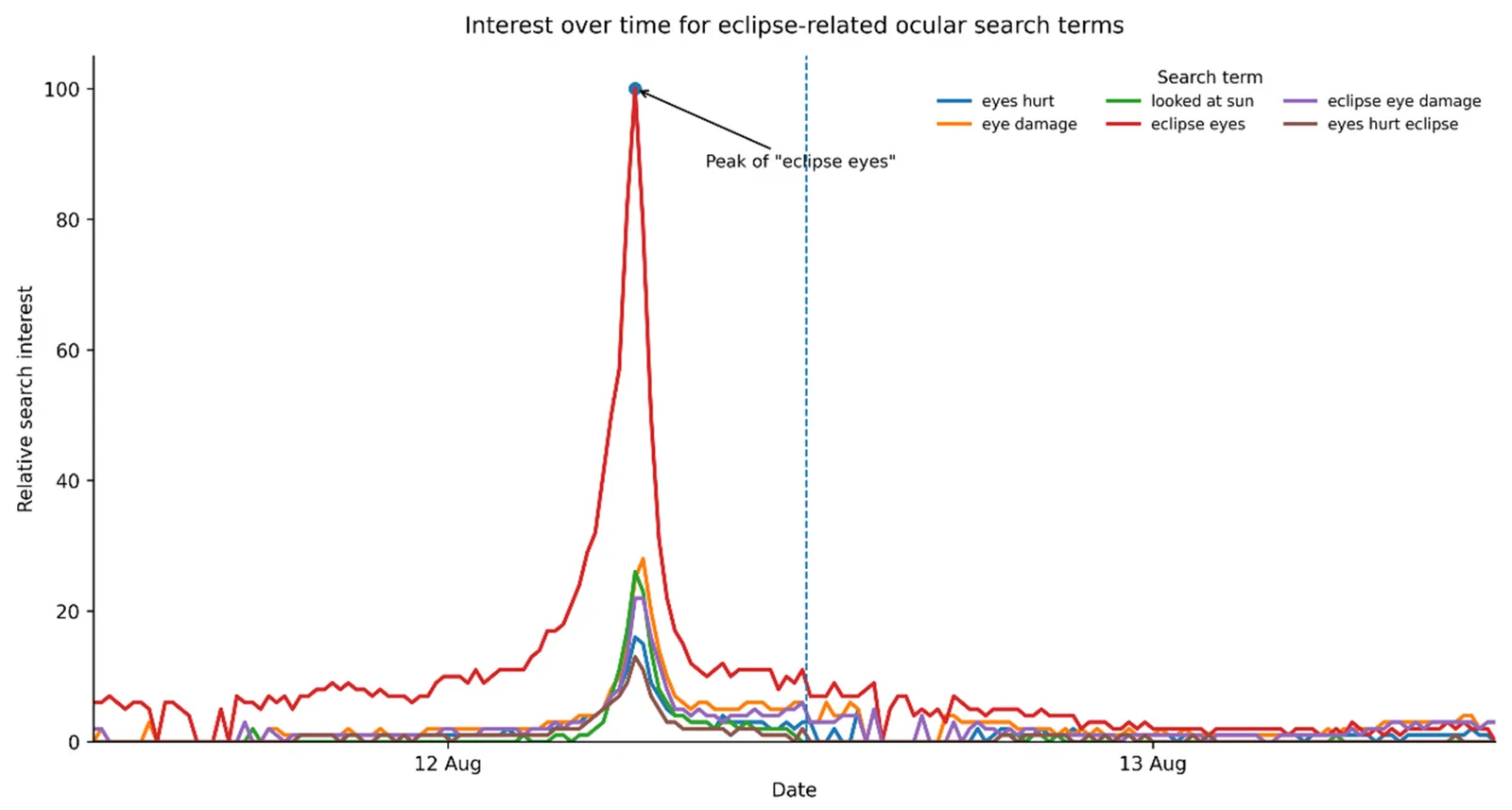
**Google Trends analysis of eclipse-related ocular search activity in the United Kingdom surrounding the 12 August 2026 partial solar eclipse.** Relative search interest for six predefined search terms—“eyes hurt”, “eye damage”, “looked at sun”, “eclipse eyes”, “eclipse eye damage”, and “eyes hurt eclipse”—during 12–13 August 2026. Google Trends values are normalised on a 0–100 scale, where 100 represents the point of greatest relative search interest within the selected comparison; “eclipse eyes” reached the peak value of 100. The dashed vertical line separates observations from 12 and 13 August. Google Trends values represent relative search interest rather than absolute search volumes and should not be interpreted as measures of symptoms, ocular injury or clinical incidence.

**Table 1:** **Patient level and eye level characteristics.**

Variable	Value
**Patient-level (n = 10)**	
Age, years; median (IQR)	37.5 (29.8–42.5)
Female sex	7/10 (70%)
Bilateral symptoms	9/10 (90%)
Intentional solar viewing	7/10 (70%)
Viewing duration < 5 s	6/10 (60%)
Viewing duration 5–30 s	2/10 (20%)
Viewing duration 31–60 s	1/10 (10%)
Viewing duration > 1 min	1/10 (10%)
Single sustained gaze	8/10 (80%)
Repeated glances	2/10 (20%)
Confirmed certified eclipse-glasses use	0/10 (0%)
Ordinary sunglasses used	1/10 (10%)
Homemade cardboard-framed viewer	1/10 (10%)
Immediate symptom onset	9/10 (90%)
Reduced or blurred vision	5/10 (50%)
Photophobia	4/10 (40%)
Dyschromatopsia	3/10 (30%)
Floaters	3/10 (30%)
OCT performed	10/10 (100%)
Ultra-widefield CFP performed	9/10 (90%)
FAF performed	9/10 (90%)
**Eye-level (n = 20)**	
Presenting VA ≥ 6/9	20/20 eyes (100%)
Presenting VA ≥ 6/6	17/20 eyes (85%)
Pinhole VA unchanged from presenting VA	20/20 eyes (100%)
Clinically apparent macular abnormality	0/20 eyes (0%)
Definite acute solar retinopathy on day-1 imaging	0/20 eyes (0%)

CFP, colour fundus photography; FAF, fundus autofluorescence; IQR, interquartile range; OCT, optical coherence tomography; VA, visual acuity.

**Table 2:** **Top and rising Google Trends queries associated with “eyes hurt” in the United Kingdom, 12–13 August 2026.**

Rank	Query	Relative Search Interest	Change
**Top Queries**			
1	eyes hurt eclipse	100	+30%
2	eclipse eyes hurt	98	+30%
3	eclipse hurt eyes	96	+30%
4	my eyes hurt	47	−4%
5	solar eclipse hurt eyes	44	+20%
6	eyes hurt solar eclipse	41	+10%
7	solar eclipse eyes hurt	41	+10%
8	my eyes hurt eclipse	29	+100%
9	does eclipse hurt your eyes	24	−20%
10	eyes hurt after eclipse	20	+650%
**Rising Queries**			
1	eyes hurt after solar eclipse	9	Breakout
2	eyes hurt after looking at eclipse	6	Breakout
3	eyes hurt after looking at sun	4	Breakout
4	eyes hurt after looking at solar eclipse	3	Breakout
5	my eyes hurt after looking at the eclipse	3	Breakout
6	eyes hurt after looking at the sun	3	Breakout
7	eyes hurt from looking at sun	2	Breakout
8	why will the eclipse hurt your eyes	2	Breakout
9	why do my eyes hurt after the eclipse	2	Breakout
10	my eyes hurt after looking at the sun	1	Breakout

Google Trends values are normalised measures of relative search interest rather than absolute search volumes. For top queries, relative search interest is scaled from 0 to 100 within the selected comparison. Breakout denotes an exceptionally large increase in relative search interest.

## Data Availability

An anonymised dataset may be made available from the corresponding author on reasonable request, subject to institutional information-governance requirements.
